# The Acceptability of Comprehensive Smoke-Free Policies to Low-Income Tenants in Subsidized Housing

**Published:** 2010-04-15

**Authors:** Linda L. Drach, Barbara A. Pizacani, Kristen L. Rohde, Stacey Schubert

**Affiliations:** Oregon Department of Human Services, Public Health Division; Multnomah County Health Department and Oregon Department of Human Services/Public Health Division, Portland, Oregon; Multnomah County Health Department and Oregon Department of Human Services/Public Health Division, Portland, Oregon; Multnomah County Health Department and Oregon Department of Human Services/Public Health Division, Portland, Oregon

## Abstract

Our objective was to evaluate the acceptability of a comprehensive smoke-free policy among low-income tenants in a group of subsidized, multiunit buildings. We conducted a mixed-methods evaluation that included questionnaires mailed to 839 tenants and follow-up telephone interviews with 23 tenants who were current, former, and never smokers. Most never and former smokers supported the policy, citing improved health, fire safety, and building cleanliness; most current smokers disliked the policy and did not follow it. Messages focusing on shared community-level concerns, accompanied by smoking cessation resources, may support the transition to smoke-free policies in subsidized housing.

## Objective

In the summer of 2009, the US Department of Housing and Urban Development began to encourage smoke-free policies in public housing to prevent secondhand smoke migration between units, many of which are inhabited by tenants particularly vulnerable to the negative health effects of secondhand smoke (eg, elderly people, children, people with chronic illnesses) (1-3). Our objective was to evaluate the acceptability of a comprehensive smoke-free policy among low-income tenants in a group of subsidized, multiunit buildings.

## Methods

On January 1, 2008, Guardian Management, LLC, the largest property management company in metropolitan Portland, Oregon, implemented a smoke-free policy for all indoor spaces and outdoor communal areas within 25 feet of buildings. We partnered with Guardian to evaluate policy acceptability in 17 subsidized buildings housing low-income, mostly elderly or disabled residents. We conducted a mixed-methods evaluation that included a questionnaire mailed to all current tenants and in-depth, qualitative interviews with 23 tenants. The evaluation was approved by the Oregon Department of Human Services' institutional review board.

In May 2008, 839 tenants received a questionnaire, cover letter, and a $2 bill as an incentive. The cover letter explained the evaluation, assured privacy and confidentiality, and promised $25 as a "thank you" for completing the questionnaire. Data were analyzed by using SPSS version 15.0 (SPSS, Inc, Chicago, Illinois).

We conducted qualitative, follow-up interviews with 23 tenants (5 current, 10 former, and 8 never smokers) who responded to the written survey. Tenants were selected by reported smoking status and to maximize variability across study buildings. Eight current smokers were selected, and the first 5 who returned an "opt-in" card were interviewed. Twenty-four nonsmokers were selected; 18 returned opt-in cards and were interviewed. Interviews were conducted by telephone in August 2008 (current smokers) and August 2009 (nonsmokers). All interviewees gave informed consent and were mailed $25 checks.

Tenant interviews were audiotaped, transcribed verbatim, and organized by using NVivo version 8.0 (QSR International, Cambridge, Massachusetts). A primary coder used methods informed by grounded theory to develop open codes, build categories through constant comparison, and develop a coding tree that was refined through ongoing discussions with the analytic team (4,5).

## Results

Eighty-two percent of tenants returned questionnaires. Most respondents were white (87%), women (69%), and aged 55 years or older (64%). Most (75%) reported at least 1 major chronic illness or disability and most were nonsmokers (39% never smokers, 35% former smokers).

Overall, 74% of tenants were "very" or "somewhat" happy with the smoke-free policy, but opinions varied by smoking status. Only 30% of current smokers were happy with the policy, compared with 85% of former smokers and 92% of never smokers (*P* < .001).

Similar themes were identified in qualitative interviews with former and never smokers. Nonsmokers praised the policy for promoting health, fire safety, and building cleanliness:

People can get really sick from being in rooms with secondhand smoke. . . . I think it's a much more healthy way to live, with the no smoking policy.

Someone could fall asleep with a lit cigarette and start a fire, so I like that that can't happen now.

The air is fresher. . . .

Smokers' primary objection was that the policy was unfair, particularly because it was implemented after their tenancy was established:

This is my home. You can't tell me what to do in my home!

I signed that contract knowing I could smoke in my apartment. Otherwise, I doubt that I would have moved in here. I would have found a different place.

Nevertheless, some smokers recognized positive aspects of the new smoke-free policy: 

I think [the policy] is good for the ones who are very rude about their cigarettes, as far as blowing smoke in front of other tenants or leaving . . . cigarette butts.

Acceptance and adherence appeared to be related. Five months after the policy was implemented, 62% of smokers reported that they did not follow the policy (50% of those happy with the policy vs 68% unhappy with it, *P* = .04).

## Discussion

Like their counterparts in private housing (6,7), most tenants in subsidized housing support smoke-free policies, but acceptance varies by smoking status. Because of low income, advanced age, or disability, and because of a limited supply of subsidized housing, residents have less freedom to move if they dislike the policies and cannot simply be given notice of eviction, as in private housing. Therefore, policy acceptance by all tenants, including smokers, matters. Approximately 2 in 3 smokers reported both unhappiness and nonadherence with the policy, a substantial enough proportion to derail successful implementation.

Smokers focused on the policy's unfairness but acknowledged its benefit to others. Messages that emphasize shared community concerns like tenant rights (including avoidance of secondhand smoke), building cleanliness, and fire safety may encourage more tenants, particularly smokers, to accept new smoke-free policies.

An aggressive focus on cessation is also needed if smoke-free policies are to be successful in this setting. Tailored approaches to cessation that take into consideration the special needs of elderly (8) and disabled smokers, including those with mental illness (9), are warranted and, like workplace cessation efforts, should build on the easy access to large, relatively stable populations who spend substantial amounts of time in a single setting (10).

Some limitations apply to this evaluation. Our response rate was high, but because we first surveyed tenants 5 months after the policy was implemented, the unhappiest tenants may have already left, thereby biasing our results. However, because tenants in subsidized housing have less mobility, the effect is probably negligible.

Smoke-free policies in subsidized, multiunit housing are urgently needed. Messages aligned with tenant values, including those of smokers, may increase acceptability and, consequently, compliance. Providing tailored cessation resources can further amplify the success of smoke-free policies and should always accompany implementation of smoke-free policies.
